# Bibliometric and visualisation analyses of gastric ulcer knowledge areas and emerging trends, 2004–2024

**DOI:** 10.3389/fmed.2025.1530835

**Published:** 2025-02-21

**Authors:** Zijian Liu, Yi Liu, Yang Li, Yuyan Sun, Xiaoxiang Song, Lu Chen, Dan Zhou

**Affiliations:** Department of Acupuncture and Tuina, Changchun University of Chinese Medicine, Changchun, Jilin, China

**Keywords:** gastric ulcer, stomach ulcers, *Helicobacter pylori*, marginal ulcer, antioxidant, gut microbiota, antibiotic resistance

## Abstract

**Background:**

Gastric ulcers are a form of peptic ulcers that present as ruptures of the mucosal lining of the stomach or the proximal intestinal lining extending beyond the muscularis mucosae, and *Helicobacter pylori* infection is one of the main causative factors of gastric ulcers. However, the growing incidence of *Helicobacter pylori* drug resistance and the emergence of specialised ulcers has necessitated continued research on gastric ulcers. This study surveyed global gastric ulcer research over the past two decades with the aim of identifying the major findings and emerging trends in the field.

**Methods:**

Bibliometric analysis was performed using the search terms ‘Gastric ulcer’, ‘Gastric ulcer disease’, ‘Gastrohelcoma’, and ‘Stomach ulcers’. Data were extracted from the Web of Science Core Database (WoSCC) and visualised using CiteSpace software.

**Results:**

The Journal of Gastroenterology had the most cited papers. The largest number of papers was from the United States. The most frequently cited keywords were ‘*Helicobacter pylori*’, ‘peptic ulcer’, and ‘gastric ulcer’.

**Conclusion:**

The field of gastric ulcer research is rapidly expanding, and the existing research is focused on preventing the occurrence of gastric ulcers, exploring the pathogenesis of gastric ulcers, and identifying new methods of treating gastric ulcers.

## 1 Introduction

Gastric ulcers are a form of peptic ulcers that present as ruptures of the mucosal lining of the stomach or the proximal intestinal lining extending beyond the muscularis mucosae (1). They are primarily characterised by periodic epigastric pain, which is usually relieved by eating or ingesting alkaline substances (2). *Helicobacter pylori* was identified as one of the main causes of peptic ulcers in 1983, necessitating the use of antibiotics to treat gastric ulcers. However, due to the excessive use of antibiotics, which has led to an increase in drug resistance in patients, the treatment of gastric ulcers and the direction of research have continued to change in recent years (3). Hooi et al. analysed research articles published over a 45-year period on the prevalence of *Helicobacter pylori* infection and deduced that more than half of the world’s population is infected with *Helicobacter pylori* (4). To facilitate clinical management and to speculate on future research trends, this study aimed to summarise and use bibliometrics to analyse research hotspots and trends in gastric ulcers.

## 2 Materials and methods

### 2.1 Data sources

The data were retrieved online through the Science Citation Index-Expanded version of the Web of Science Core Collection (WoSCC). All data were obtained on 27 January 2024 and spanned the period from 2004 to 2024.

### 2.2 Inclusion criteria

**Figure 1:**
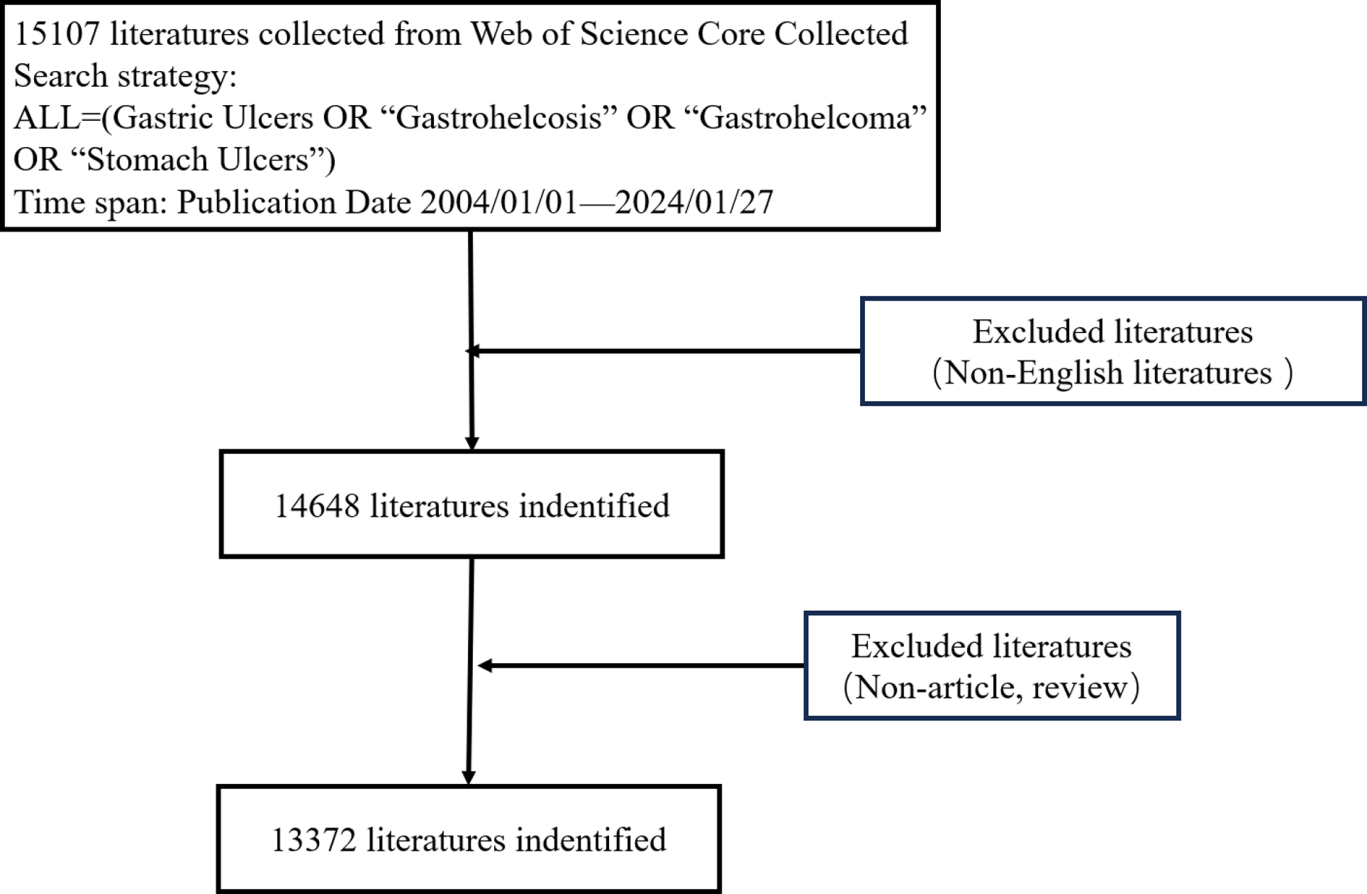
Procedures of bibliometric analysis.

The following terms were searched in the topic: ‘Gastric Ulcers’ or ‘Gastrohelcosis’ or ‘Gastrohelcoma’ or ‘Stomach Ulcers’; the language choice was English; and only original articles and reviews were included. Two independent investigators reviewed the titles and abstracts, and studies unrelated to gastric ulcers were excluded. After reviewing titles and abstracts, 13,372 publications were retained. The detailed retrieval process is shown in Figure 1.

### 2.3 Analysis tool

Bibliometrics is a method used to assess research in a target area through an objective and reliable structured analysis of large amounts of information (5, 6). CiteSpace is a scientific software that is usually used to summarise existing results in a target area and to identify future research directions by analysing research trends in the literature (7). CiteSpace combines bibliometrics and data mining algorithms with visual analytics to analyse and present potential knowledge and research hotspots in the form of images (8). It provides information about authors, countries/regions, institutions, keywords, journals, etc., and performs co-occurrence, co-citation, and cluster analyses on related information in image form to visualise research trends and patterns and to mine the correlations between the information in each part of the literature (9). It can either comprehensively reflect the research status of a discipline or explore a discipline from different directions, helping researchers quickly and accurately understand the research hotspots and research trends in the field and so on. VOSviewer is an application used to build and view bibliometric maps (10). It analyses and summarises data and information to present an overview of the research in the field in the form of graphs, thereby objectively presenting the current status of research in a certain field. Therefore, with the help of CiteSpace and VOSviewer visualisation and analysis software, this study collected, analysed, and processed literature information in related fields, drew images and tables related to this topic, and sorted the current status of research on gastric ulcers and hotspot issues, which can be used as a reference for treatment and future research.

Based on the adopted data and statistical purposes, corresponding visualisation graphs can be generated in CiteSpace, where different nodes and links exist in different graphs, and nodes with high centrality are indicated as research hotspots or turning points in the field (11). We conducted a relevant literature search of the WoSCC and exported the retrieved data in a plain text format; the export included complete records and references. The exported file was named download_XXX.txt and was imported into CiteSpace 6.2. R7 for bibliometric and visualisation analyses. Cluster analysis of co-occurring keywords was performed using CiteSpace to reveal major themes. The contour function (Sihouette, S) is commonly used to assess clustering, which is generally considered reasonable if S > 0.5, and S > 0.7 indicates a high degree of homogeneity among cluster members, suggesting that the clustering results are meaningful.

In this study, the data were time-sliced in years, the node filter was g-indexed, and the k value was set to five.

## 3 Results

### 3.1 Visual mapping of publications by year

**Figure 2:**
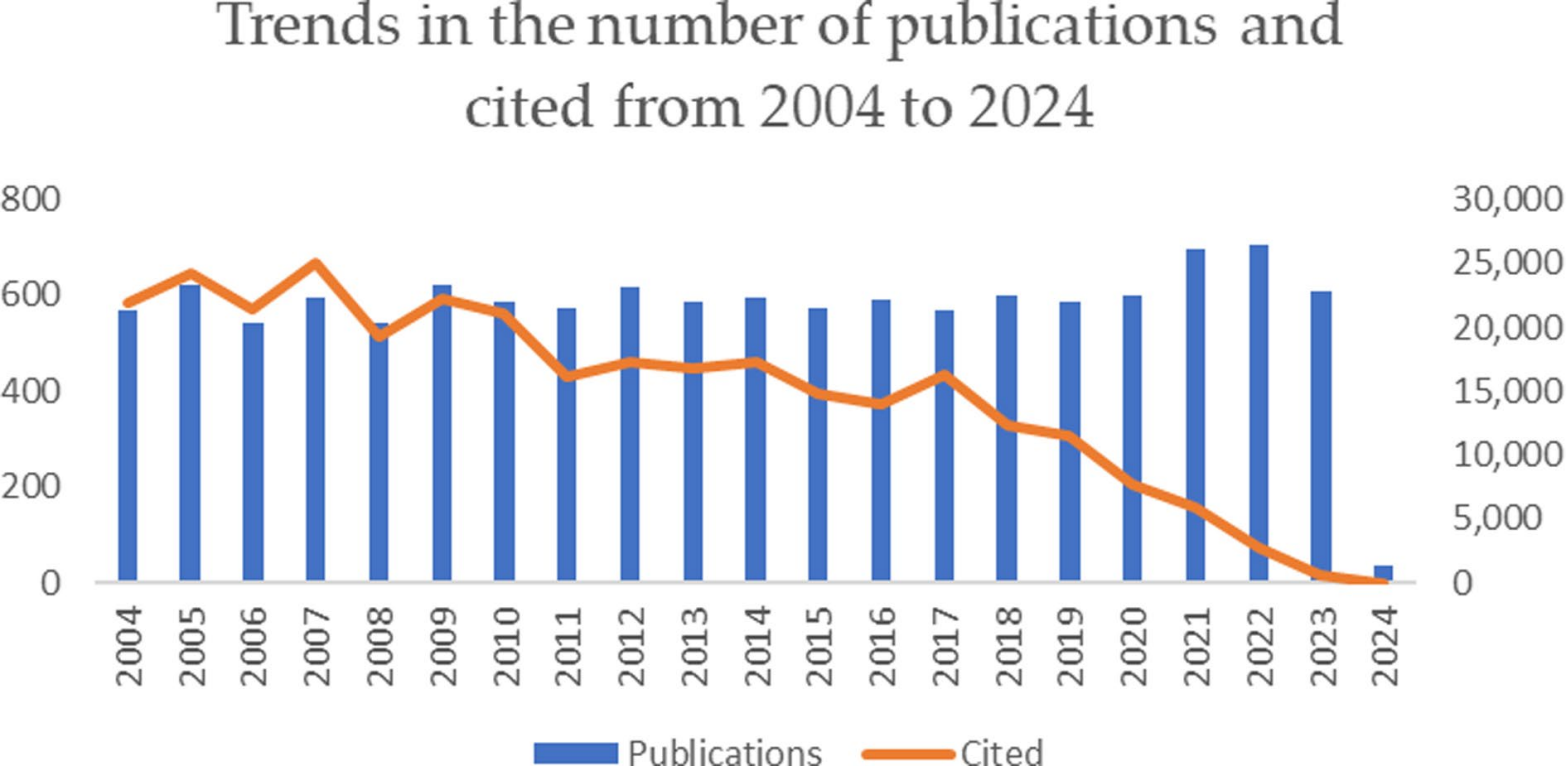
Trends in the number of publications and cited from 2004 to 2024.

After screening, we downloaded 13,372 publications from the WoSCC database. Figure 2 shows a bar dash plot of the number of publications and citation frequency for the topic of gastric ulcers from 2004 to 2024.

### 3.2 Visual mapping of national and institutional publications

**Figure 3:**
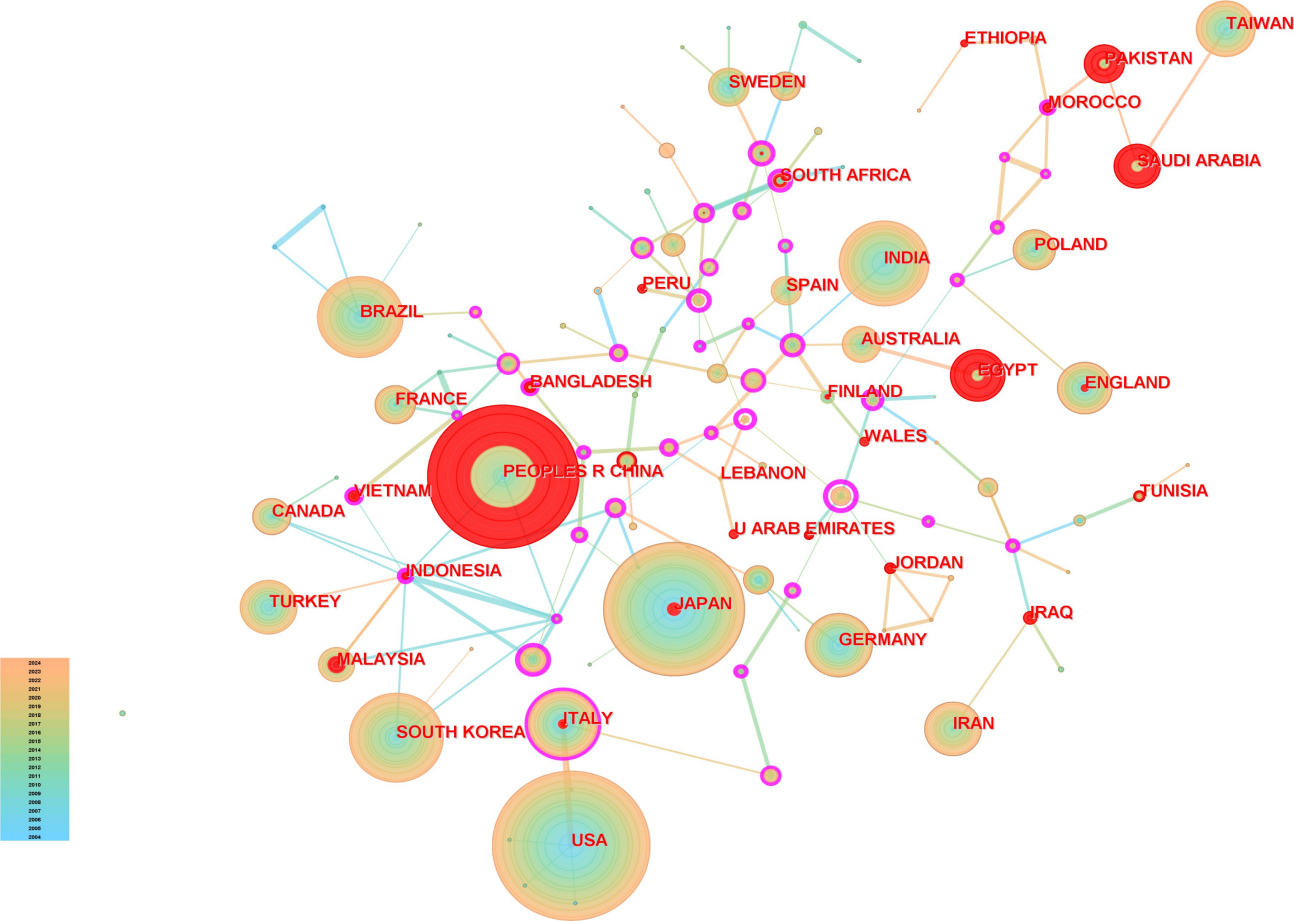
Analysis of country cooperation.

Figure 3 shows the visualisation and analysis of the country collaboration network, which generated 112 nodes and 145 links, respectively. According to the statistics, the eight countries with the highest number of publications published a total of 9,208 articles (68.9%). When analysing country centrality, Russia was found to have the highest mediator centrality (0.89; Table 1).

**Table 1:** Analysis of the number of articles sent by country.

Rank	Publication	Country	Rank	Centrality	Country
1	1,882	USA	1	0.89	Russia
2	1,762	China	2	0.84	Colombia
3	1,595	Japan	3	0.66	Chile
4	771	India	4	0.53	Hungary
5	747	South Korea	5	0.4	Ireland
6	662	Brazil	6	0.37	Cameroon
7	496	Italy	7	0.37	Bhutan
8	493	Germany	8	0.36	Czech Republic

**Figure 4:**
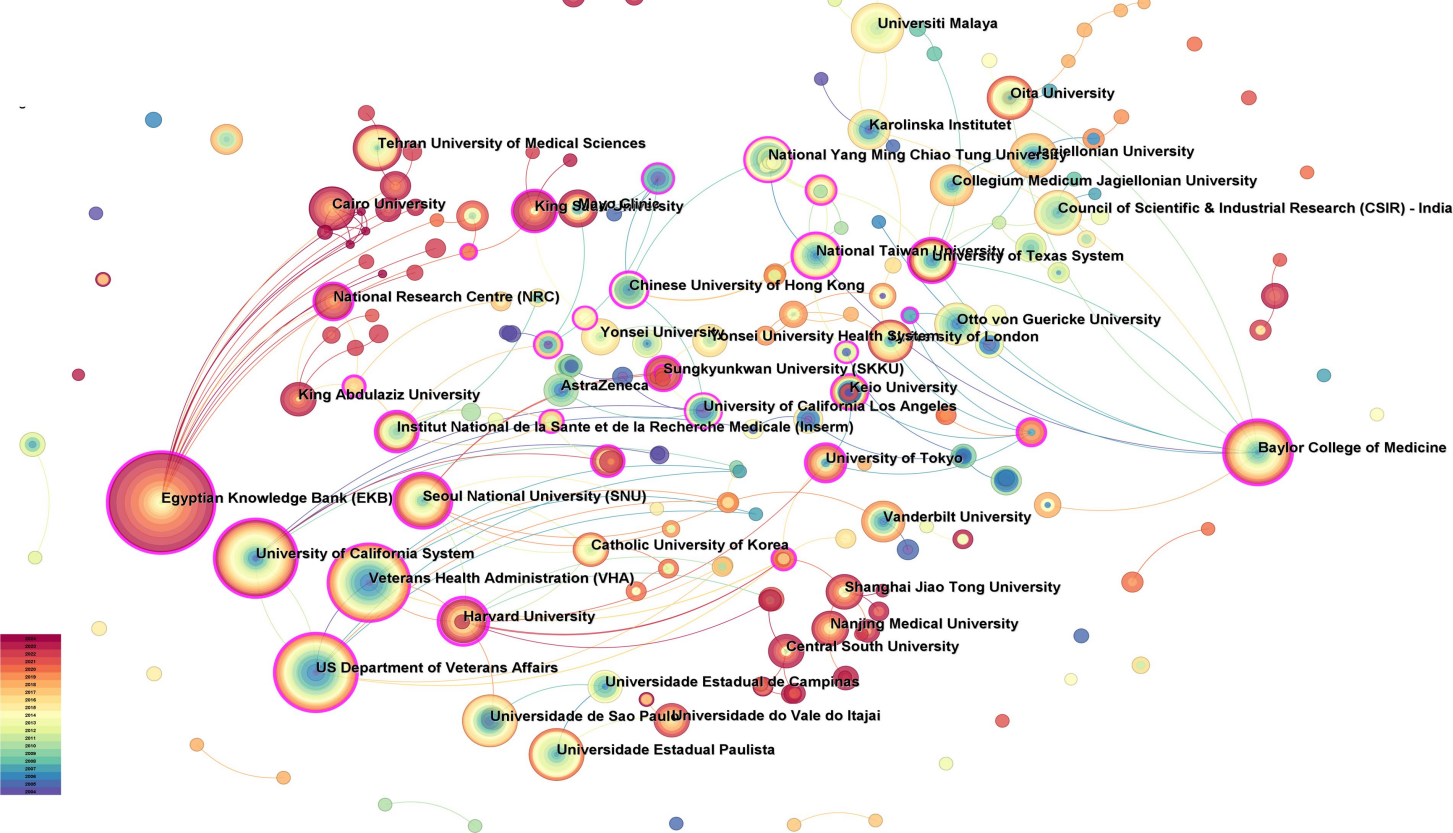
Analysis of institution cooperation.

The analysis of institutions and agencies generated 235 nodes and 259 links (Figure 4), and the top 10 institutions and agencies published 1,524 articles (11.4%), with the top five being the Egyptian Knowledge Bank, University of California, US Department of Veterans Affairs, Veterans Health Administration, and Baylor College of Medicine (Table 2).

**Table 2:** Analysis of the volume of publications by institutions and agencies.

Count	Centrality	Institution
322	0.24	Egyptian Knowledge Bank (EKB)
201	0.29	University of California System
201	0.24	US Department of Veterans Affairs
201	0.15	Veterans Health Administration (VHA)
148	0.31	Baylor College of Medicine
104	0.07	Universidade de São Paulo
95	0.03	Universidade Estadual Paulista
94	0.23	Seoul National University (SNU)
80	0.04	Universiti Malaya
78	0.09	Oita University

### 3.3 Visual mapping of journal citations

**Figure 5:**
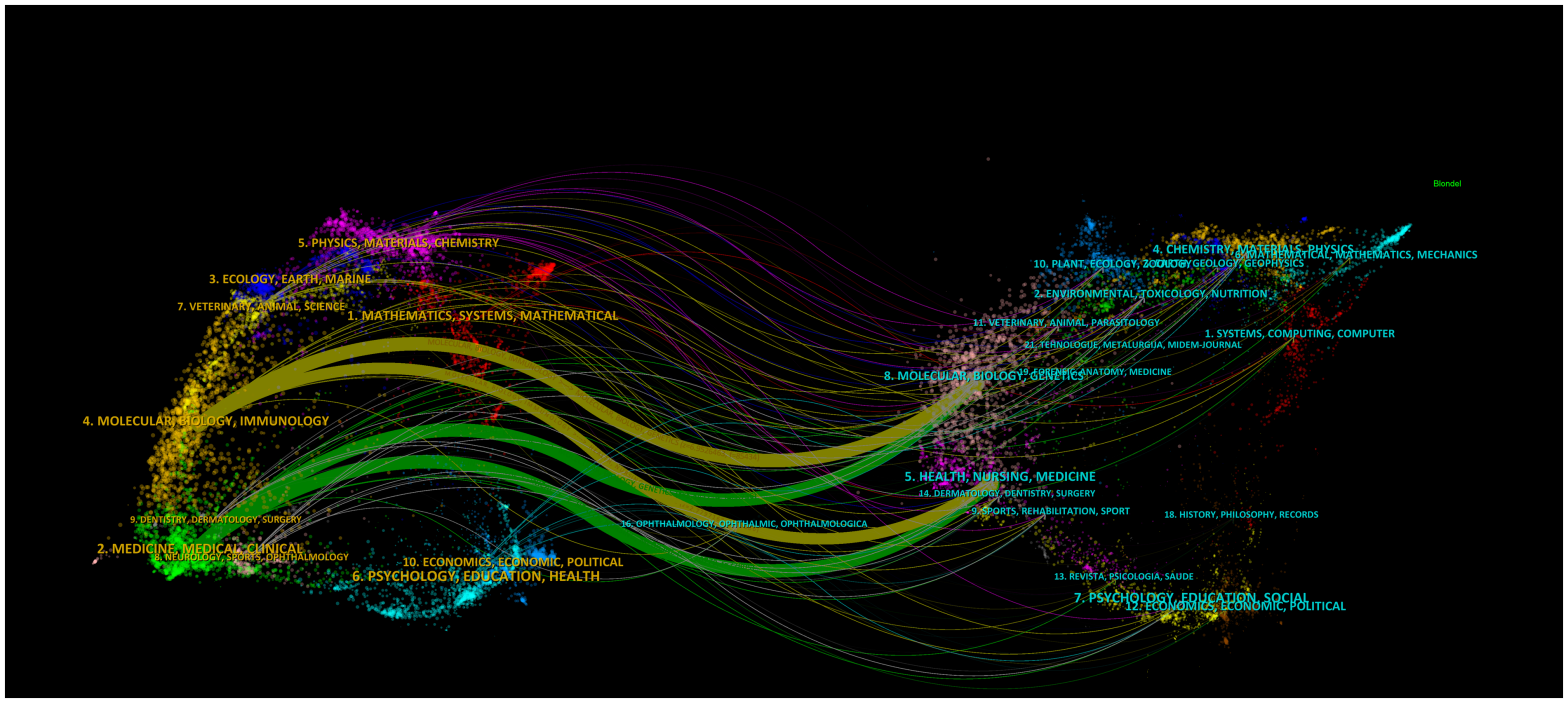
Dual-map overlay of journals.

Figure 5 shows a bi-mapped overlay mapping of journals, representing the distributional relationship between citing and cited journals as well as the development of relationships between different disciplines (12). The citing journals are located on the left side; the cited journals are located on the right side; the coloured paths indicate the correlation between citations; and the coloured nodes represent the clustering of different disciplines.

### 3.4 Visual mapping of author posting volume

**Figure 6:**
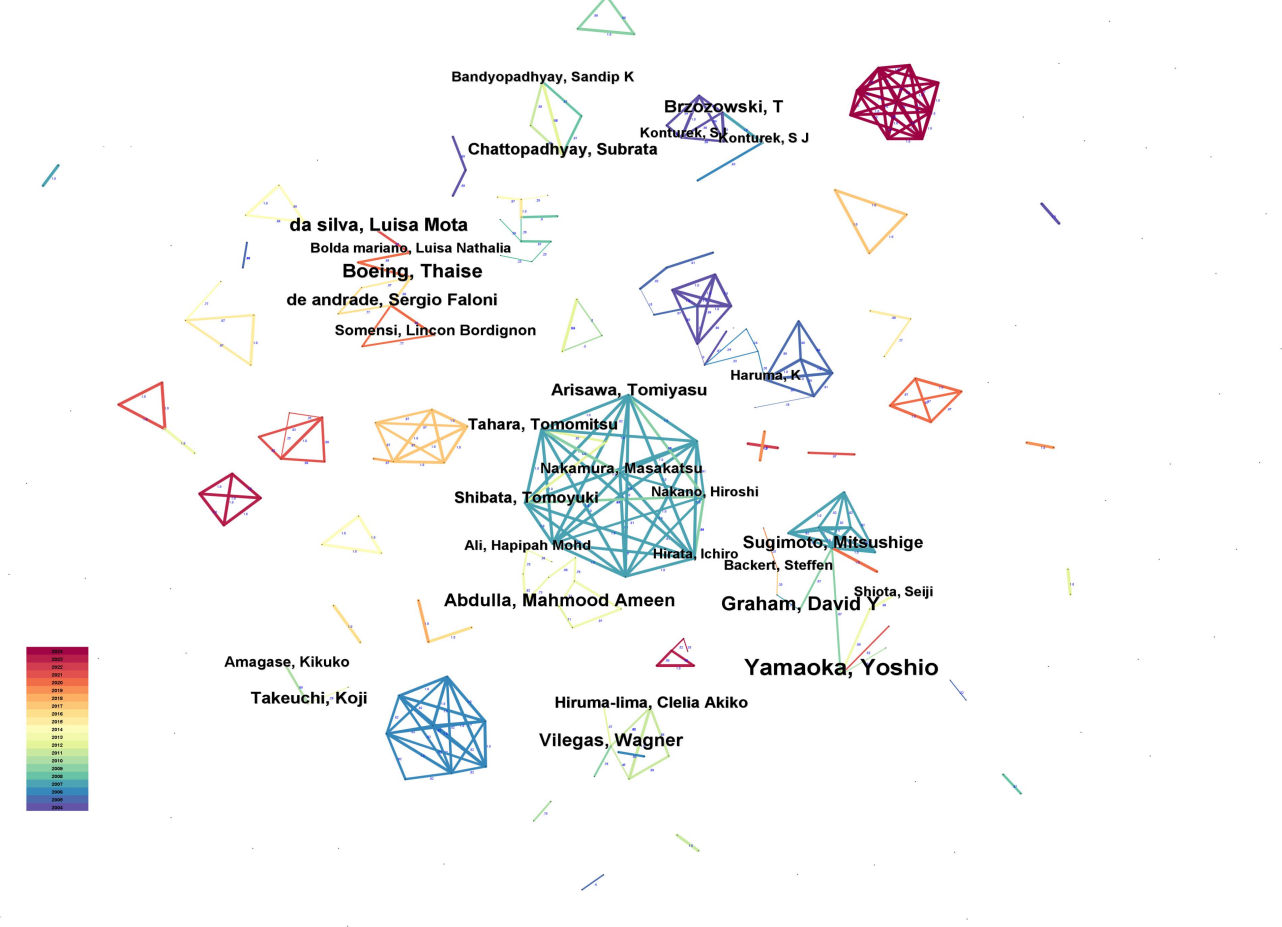
Analysis of author cooperation.

Figure 6 illustrates the author network generated by CiteSpace, where the connecting lines between nodes signify the collaborative relationships among authors. The thicknesses of these lines denote the frequency of collaboration, with thicker lines indicating greater cooperation. Complementing this visual representation, Table 3 lists the top ten authors with the highest number of publications, who collectively contributed 442 papers (3.3% of the total publications).

**Table 3:** Analysis of the number of publications by authors.

Rank	Count	Author
1	81	Yamaoka, Yoshio
2	48	Graham, David Y
3	46	Boeing, Thaise
4	45	da silva, Luisa Mota
5	43	Abdulla, Mahmood Ameen
6	41	Vilegas, Wagner
7	37	Brzozowski, T
8	34	Sugimoto, Mitsushige
8	34	de andrade, Sergio Faloni
9	33	Takeuchi, Koji
10	31	Arisawa, Tomiyasu

### 3.5 Visual mapping of co-cited references

**Figure 7:**
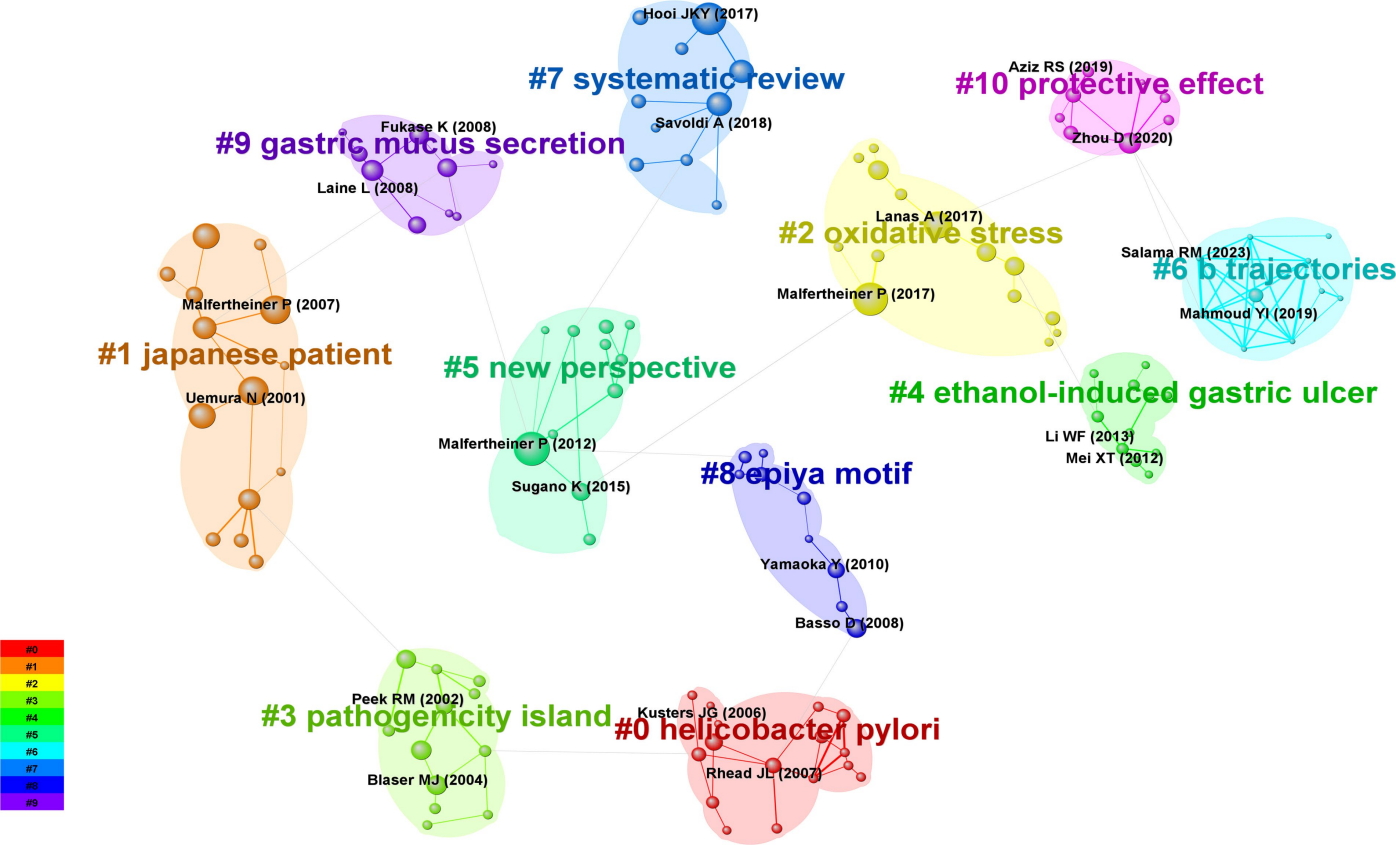
Cluster analysis of co-cited articles.

Table 4 shows the top 10 studies related to gastric ulcers, which were cited 1,339 times. The rise, growth, and decline of a research field can be observed by clustering co-cited literature, with more documents in a cluster indicating greater importance of the clustered field. Figure 7 shows the sequence plot of the top 10 co-cited documents plotted as nodes for each time slice.

**Table 4:** Analysis of the number of literature co-citations.

Panking	Title	Author	Citations
1	Management of *Helicobacter pylori* infection-the Maastricht IV/ Florence Consensus Report	Malfertheiner, P	192
2	Management of *Helicobacter pylori* infection-the Maastricht V/Florence Consensus Report	Malfertheiner, P	182
3	Global Prevalence of *Helicobacter pylori* Infection: Systematic Review and Meta-Analysis	Hooi, James K. Y.	178
4	*Helicobacter pylori* infection and the development of gastric cancer.	Uemura, N	135
5	Current concepts in the management of *Helicobacter pylori* infection:: the maastricht III consensus report	Malfertheiner, P	130
6	Peptic ulcer disease	Lanas, A	120
7	Current concepts in the management of *Helicobacter pylori* infection -: The Maastricht 2–2000 Consensus Report	Malfertheiner, P	106
8	Medical progress:: *Helicobacter pylori* infection	Suerbaum, S	105
9	ACG Clinical Guideline: Treatment of *Helicobacter pylori* Infection	Chey, WD	96
10	Prevalence of Antibiotic Resistance in *Helicobacter pylori*: A Systematic Review and Meta-analysis in World Health Organisation Regions	Savoldi, A	95

### 3.6 Visual mapping of keyword frequencies

Keyword frequency reflects research hotspots in a field, and high-centrality keywords represent milestones for advancing research in the field. Table 5 lists the top 10 high-frequency and high-centrality keywords.

**Table 5:** Analysis of keywords centrality.

Rank	Count	Keyword	Rank	Centriality	Keyword
1	3,227	*helicobacter pylori*	1	1.13	vaca
2	1,765	peptic ulcer	2	1.11	antioxidant
3	1,465	gastric ulcer	3	1.08	population
4	1,358	infection	4	1.02	3′ region
5	1,200	ulcer	5	1	gastric epithelial cells
6	1,166	risk	6	0.99	pathogenicity island
7	1,161	gastric cancer	7	0.97	inflammation
8	914	duodenal ulcer	8	0.97	vacuolating cytotoxin
9	821	expression	9	0.96	nf kappa b
10	765	rat	10	0.96	increased risk

**Figure 8:**
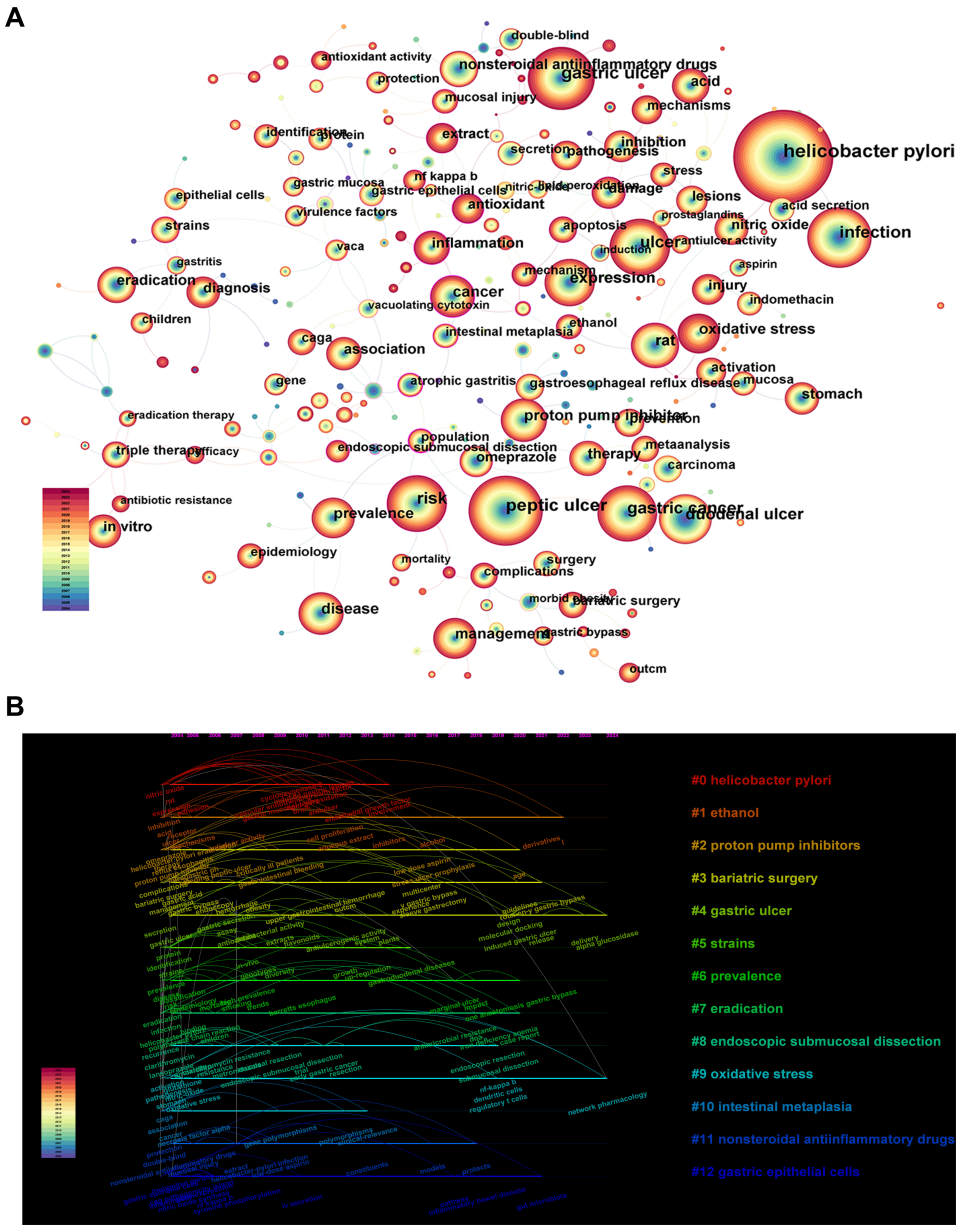
**(A)** Analysis of keywords frequency. **(B)** Analysis of keyword clustering timeline.

This field of research focuses on high-frequency keywords. High-centrality keywords reflect the status and influence of the corresponding content in the research field whereas co-occurring keywords extracted from the selected literature can reflect research hotspots and development trends in a certain field. Figure 8A shows the 242 symbiotic keywords extracted from the 13,372 manuscripts included in this study

Figure 8B shows a timeline plot of keyword clustering, where the 13 cluster names labelled # represent basic knowledge in the field of gastric ulcer research and its evolution over time, and the connecting lines in the figure indicate that two keywords occurred simultaneously in the same article. The mean contour value (Sihouette, S) is typically used to evaluate the clustering. In general, clusters are considered reasonable if S > 0.5, while an S value of 0.7 implies that the clusters are highly plausible. We obtained 13 clusters with cluster contour values greater than 0.7, indicating that the results are highly plausible.

### 3.7 Visual mapping of keyword occurrence

**Figure 9:**
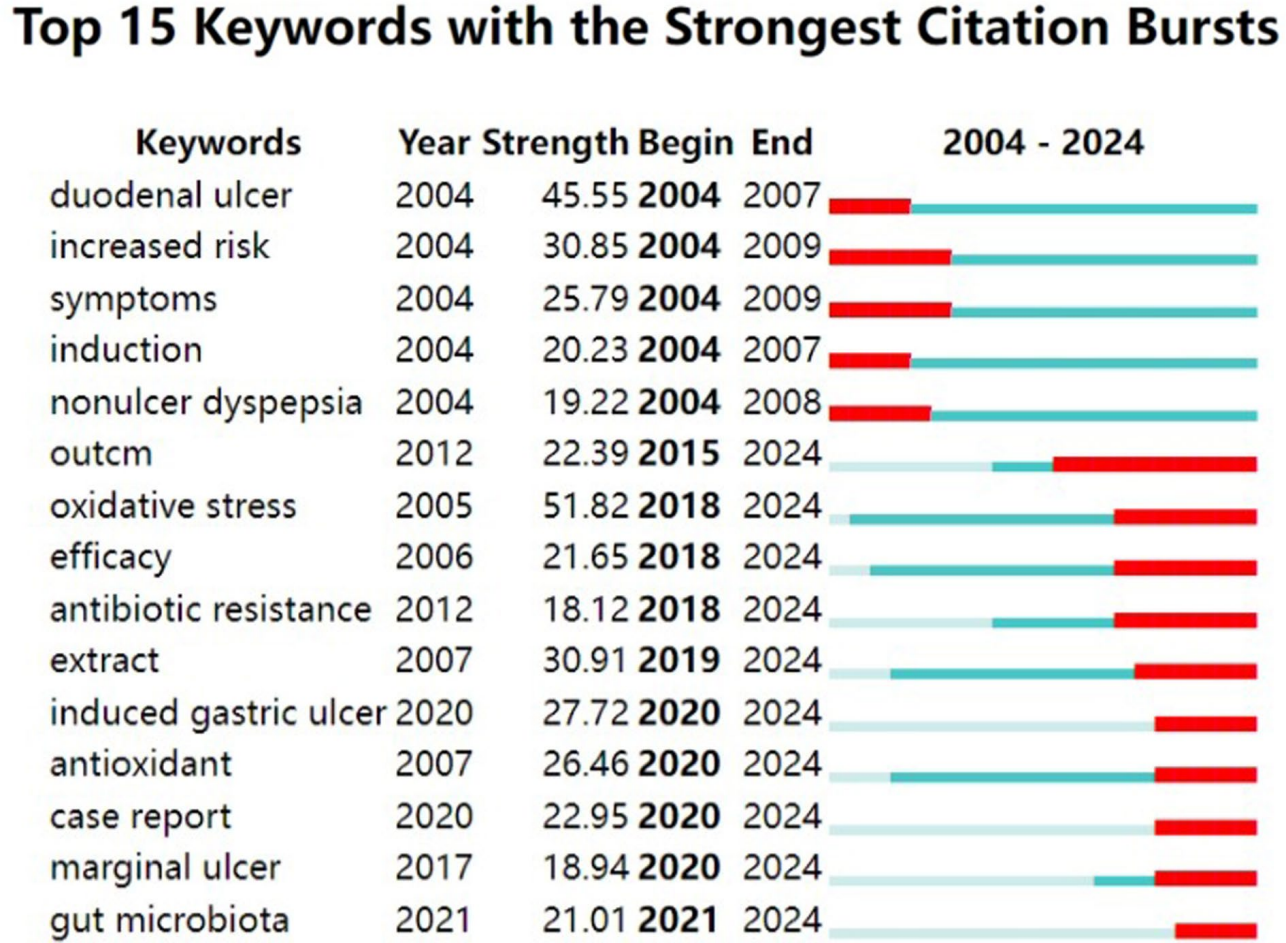
Analysis of keyword with the strongest citation bursts.

Explosive keywords reflect the research frontiers in different periods. Figure 9 shows the 15 keywords with the highest explosion rates between 2004 and 2024. The red line indicates the time period when the keywords exploded, and the blue line indicates the time interval (13).

## 4 Discussion

The analysis of the papers showed a dynamic and balanced trend of gastric-ulcer-related papers from 2004 to 2020, with an average of 585.88 papers per year. The number of citations for gastric-ulcer-related articles showed a slowly decreasing trend, indicating that many articles have not yet been cited. The combination of these two findings indicates continuing interest in the field of gastric ulcer research and that a large number of articles have research potential.

An analysis of the countries and institutions showed that the top 10 countries engaged in gastric ulcer and related research yielded 9,208 articles, accounting for 68.9% of the total number of articles, with the United States, China, Japan, India, and South Korea contributing 1,820, 1,762, 1,595, 771, and 747 articles, respectively. While these findings indicated that these countries are at the forefront in this field, when analyses based on country centrality were carried out, Russia exhibited the highest intermediate centrality (0.89), trailed by Colombia (0.84), Chile (0.66), Hungary (0.53), and Ireland (0.40). These countries with high centrality possess significant bridging potential in the field of gastric ulcer research, indicating that international collaborative research programmes should be actively pursued with them for the complex ulcer pathogenesis. Analysis of the national cooperation network showed active cooperation among Asian countries and strong partnerships between the United States and European countries. Among the five countries with the highest intermediary centrality, Russia, Colombia, Chile, and Hungary had collaborative relationships, whereas India showed a collaborative relationship with Ireland. In terms of institutions and agencies, the top 10 institutions and agencies published a total of 1,524 articles (11.4%), with the top five being Egyptian Knowledge Bank (332), University of California (201), US Department of Veterans Affairs (201), Veterans Health Administration (201), and Baylor College of Medicine (148). Two of the five institutions listed above are veteran-related, indicating that research on gastric ulcers could be actively carried out in collaboration with these or other veteran-related institutions. The Egyptian Knowledge Bank showed frequent collaborations with other institutions, such as the University of California, the US Department of Veterans Affairs, and the Veterans Health Administration. Research institutions with a high number of publications utilise a dense network of collaborations and often collaborate with other institutions. By summarising the national and institutional collaborative networks, we could see that the United States has an important position in the field of gastric ulcer research. Most countries were more independent and less collaborative with each other, and a mismatch was observed between countries with a high frequency of gastric ulcer research and countries with a central role in the field of gastric ulcer research, highlighting the potential for collaborative research between these countries in the future. The number of publications related to gastric ulcer research fluctuated annually. However, overall, this topic has remained a popular area of research, and the number of publications maintained a dynamic balance.

In the citation relationships between journals, the citing journals represent the frontiers of knowledge in the field of research, and the cited journals represent the knowledge base of the field of research. We identified four main citation paths by analysing the journal citations—the “Molecular, Biological, Genetics” domain and the “Health, Nursing, Medicine” domain were merged into the “Molecular, Biological, Immunology” domain and the “Medical, Medical, Clinical” domain. The evolving focus in gastric ulcer research reflects shifting scientific perspectives. While molecular and biological dimensions remain under investigation, current studies emphasise immunological connections and clinical implications more strongly than earlier works.

An analysis of the authors shows that the author with the highest number of publications was Yamaoka, Yoshio (81 articles), followed by Graham, David Y (48 articles), Boeing, Thaise (46 articles), da silva, Luisa Mota (45 articles) and Abdulla, Mahmood Ameen (43 articles). Highly productive authors typically have stable collaborative relationships with other authors. Based on Price’s Law, the core author’s formula is as follows:


M≈0.749√Nmax

Equation 1, Price’s Law.

Where M is equal to the threshold for the number of papers published by the core authors, while Nmax denotes the number of papers published by the author with the highest number of published papers during the analysis period. Authors who publish more than M papers are considered to be core authors, and when the total number of published papers by the core authors reaches 50% of the total number of issued papers, it indicates the formation of a core group of authors (14). Yamaoka and Yoshio were the most published authors in the network; hence, the Nmax was 81. Thus, M was equivalent to ≈6.741, implying that authors publishing more than six papers were core authors. In this study, 55,156 authors were selected, of which 919 authors had more than six papers, and the number of papers published by these core authors accounted for 11.5% of the total number of papers, indicating that a core group of authors has not yet been formed in the field of gastric ulcers and related research. Furthermore, when analysing coauthors and co-citations, we found that references with a high frequency of co-citations were not from prolific authors, indicating the need for greater collaboration among authors to promote progress in related fields and improve the quality of the studies.

Analysis of the co-cited literature revealed that the most co-cited reference was a study by Malfertheiner et al., which explored three topics related to *Helicobacter pylori* infection: indications and contraindications for diagnosis and treatment, diagnostic tests and treatment of infection, and prevention of gastric cancer and other complications (15). The second-most co-cited reference was an updated guideline for *Helicobacter pylori* infection and its related clinical manifestations and management by Malfertheiner et al., which built on previous research and provided ideas for new clinical research in this area (16). An analysis of the global prevalence of *Helicobacter pylori* associated with *Helicobacter pylori* published by Hooi et al. was the third most co-cited article. By analysing 184 studies from 62 countries, the authors of that study inferred that the prevalence of *Helicobacter pylori* infection varied widely across various regions of the world and hypothesised that more than half of the global population is infected with *Helicobacter pylori* (4). By analysing the clustering of co-cited literature, we found that the research article on #pathogenicity island published by El-Omar was widely cited since 2000, with a total of 55 citations (17). Next, #Japanese patient appeared in a large number of cited articles, among which the studies by Uemura, Malfertheiner, and Suerbaum published in *NEW ENGL J MED, ALIMENT PHARM THER* and *NEW ENGL J MED* were the most highly cited articles (18–20). #*Helicobacter pylori* was heavily cited in articles from 2004 to 2009, and related articles such as #new perspective and #epiya motifs have been important research topics in the field of gastric ulcers since 2009. Studies on oxidative stress and its trajectories since 2013 have provided important research information and assistance to the field.

Keywords represent a high-level summary of the research topic of an article and can be used to quickly understand the main research content of an article. The analysis of keywords can be used to identify important issues in the research field and to predict future research trends. The node size of the keyword co-occurrence graph represents keyword frequency. According to the statistics provided by the CiteSpace software (Figure 8A), ‘*Helicobacter pylori*’ was the largest node in the keyword network, which indicates that most of the research conducted in the field of gastric ulcers is related to *Helicobacter pylori*. The next-highest ranked frequencies were ‘Peptic ulcer’, ‘Gastric ulcer’, and ‘Infection’, indicating that these items have the next-highest research statuses in gastric ulcer-related research. In the keyword network, the purple part of the node indicates that the target mediates centrality. Nodes with high mediocentricity are usually considered hotspots or turning points in research content (21). ‘Vaca’ had the highest mediated centrality in the whole keyword network, followed by ‘Antioxidant’, ‘Population’, ‘3’ region’ and ‘Gastric epithelial cells’, suggesting that these themes play crucial roles in connecting and transmitting the entire network of relationships.

### 4.1 Research hotspots for gastric ulcers

Clustering of keywords can be used to summarise a topic, and the analysis of keyword clustering can be used to identify hot topics in a research field. We summarised the 15 clusters generated and found that the hot topics of research in the field of gastric ulcers included the following three aspects: predisposing factors, pathogenesis, and diagnostic and therapeutic options for gastric ulcers.

The pathogenesis of gastric ulcers is complex, and many risk factors can lead to the development of this disease. *Helicobacter pylori*, ethanol, bariatric surgery, and non-steroidal anti-inflammatory drugs (NSAIDs) in the keyword clustering were common factors that contributed to gastric ulcers, and significant temporal and geographic differences were observed in these factors. The prevalence of many gastrointestinal disorders does not remain constant but increases and decreases (22). A European study found an underlying birth cohort trend in gastric ulcers, and this emergence is likely shaped by an underlying trend in *Helicobacter pylori* infection (23). However, the incidence, hospitalisation, and mortality rates of gastric ulcer-related diseases have decreased worldwide over the past few decades (22, 24, 25).

Although the clinical manifestations of most gastric ulcers are similar, their pathogenesis appears to be dramatically different owing to different pathogenic factors. For example, inflammation associated with *Helicobacter pylori* infection can present with two different manifestations, either too little or too much gastric acid secretion, culminating in different types of ulcers (26). These effects can be mediated by cytokines that inhibit mural cell secretion, or by the activation of calcitonin gene-related peptide (CGRP) sensory neurones coupled with growth inhibitory hormone stimulation and inhibition of histamine secretion to acutely inhibit gastric secretion and form gastric ulcers (22, 27). Another important factor contributing to ulcer development is that, unlike *Helicobacter pylori*, which colonises the stomach, NSAIDs can damage the gastroduodenal mucosa via a holistic mechanism. Inhibition of cyclooxygenase 1 (COX-1), a prostaglandin-derived enzyme, is thought to be the main mechanism by which NSAIDs damage the mucosa (28). Nevertheless, while *Helicobacter pylori* infection, NSAID use, or post-bariatric surgery complications and stress are distinct risk factors that contribute to gastric ulcers through different mechanisms, their effects ultimately manifest as damage to the gastric epithelium (29–32). Therefore, the study of injury mechanisms, including gastric epithelial cell injury, has become a focus in gastric ulcer research.

Since the pathogenesis of gastric ulcers is determined by the pathogenic factors, the therapeutic strategies are dependent on the pathogenesis. For example, antibiotic treatment is the first choice for gastric ulcers caused by *Helicobacter pylori* infection. In contrast, NSAID-mediated gastric mucosal injury is often treated with drugs that inhibit gastric acid secretion and neutralise luminal acidity, effectively preventing gastric ulcers (33, 34). However, several issues have emerged over time. Declining eradication rates of *Helicobacter pylori*, increasing rates of multidrug resistance in children, adverse reactions associated with the extensive use of proton pump inhibitors (PPIs), a gradual increase in the number of idiopathic ulcers, and a number of other factors have prompted an urgent search for new therapeutic approaches (35–37).

### 4.2 Trends in gastric ulcer research

Breaking keywords represent the research frontiers of a discipline over time, and an analysis of these keywords can yield predictions and indicate future research trends for the corresponding discipline. We found a clear temporal trend in the research on gastric ulcers. In the early stage of research, the diagnosis and classification of gastric ulcers were studied, such as ‘Peptic ulcer’, ‘Duodenal ulcer’, ‘Increased rise’ and so on. Later, researchers conducted cellular-level studies, shifting their focus to pathogenic factors and cellular damage, such as ‘*Helicobacter pylori’* and ‘gastric epithelial cells’. More recently, researchers have focused on studying pathogenic factors and cellular damage through studies related to pathogenesis, therapeutic means and sequelae of treatment, such as ‘Antioxidant’, ‘Marginal ulcer’, ‘Gut microbiota’, and ‘Antibiotic resistance’.

#### 4.2.1 Antioxidants

Reactive oxygen species (ROS) are the products of normal cellular metabolism, and the gastrointestinal tract is a major source of ROS. Low and moderate amounts of ROS can kill pathogens and promote wound healing and tissue repair; however, when stimulated by external sources, such as smoking, alcohol, and long-term treatment with NSAIDs, excess ROS can lead to oxidative tissue damage and disruption of normal cellular homeostasis (38). When specific substances or pathogens are ingested, the body will activate epithelial cells, polymorphonuclear neutrophils (PMN), and macrophages to produce inflammatory cytokines and other mediators leading to oxidative stress, and oxidative stress can lead to a variety of gastrointestinal disorders, including gastric and duodenal ulcers (39). Tumour necrosis factor-*α* (TNF-α) is an important regulator of a complex signalling network, where mitochondrial or non-mitochondrial production of ROS and TNF-α interact with each other in a positive feedback loop (40, 41), potentially causing apoptosis, destruction of the gastrointestinal barrier, and the eventual formation of ulcers (42–44). Antioxidants are molecules that inhibit or delay cellular damage by oxidising themselves and removing free radicals from tissues (38, 45). Therefore, the proper use of antioxidants is one of the most important strategies to treat gastric ulcers. Antioxidants can be broadly categorised into two groups: antioxidant enzymes and low-molecular-weight antioxidants. Antioxidant enzymes such as superoxide dismutase (SOD), catalase (CAT), and glutathione peroxidase (GPx) play crucial roles in converting metabolites into less harmful substances, such as hydrogen peroxide and water. In contrast, low-molecular-weight antioxidants function primarily by terminating the chain reactions initiated by free radicals. Key examples of these compounds include vitamins C and E, carotenoids, and flavonoids (46). Dietary intake is a crucial source of antioxidants for the human body. A Korean study reported that a phytochemical-rich diet reduced the risk of gastric ulcers (47). In addition, ongoing molecular and botanical research is expanding the variety of available antioxidants. For instance, Xie, Y.J. et al. demonstrated that Dioscoreae Rhizoma significantly increased the levels of antioxidant enzymes such as GPx, CAT, and SOD, while concurrently reducing malondialdehyde (MDA) levels and inhibiting nuclear factor (NF)-κB phosphorylation in ethanol-induced gastric ulcers (48). Similarly, Bhattacharya et al. found that *Piper betel* exhibits a comparable antioxidant mechanism (49). Additionally, Akram et al. discovered that *α*-bisabolol, found in chamomile, significantly inhibits ROS and stimulates the antioxidant system. Medicinal plants, such as Glycyrrhiza, exhibit comparable effects (50, 51). Notably, depending on the dose used, antioxidants such as resveratrol, *β*-carotene, and lycopene can have diametrically opposite antioxidant and pro-oxidant properties (52–54).

#### 4.2.2 Marginal ulcers

According to the World Health Organisation, non-communicable diseases (NCDs) account for >70% of premature deaths worldwide, and obesity is a major factor in the development of NCDs (55). Obesity directly contributes to the development of cardiovascular risk factors, including dyslipidaemia, type 2 diabetes, hypertension, and sleep disorders (56). Bariatric treatments usually include surgical and nonsurgical treatments; however, a large body of evidence suggests that surgical treatments have better weight loss outcomes and long-term efficacy (57). A meta-analysis of bariatric surgeries found that surgically treated obese patients had a lower all-cause mortality rate than non-surgically treated patients (58). Currently, approximately 49% of bariatric surgeries involve laparoscopic Roux-and-gastric bypass (LRYGB). The procedure is relatively safe and effective; however, some long-term complications may occur years after the surgery. One of these complications manifests as ulcers at the gastrojejunal anastomosis site, also known as marginal ulcers (59). Most patients with marginal ulcers present with manifestations similar to those of gastric ulcers, such as chronic abdominal pain, heartburn, reflux, and nausea. However, some patients do not show any symptoms despite the detection of marginal ulcers on examination, and the absence of warning signs before haemorrhage or perforation can endanger the patients’ lives (60, 61). According to statistics, the incidence of marginal ulcers after gastric bypass is approximately 25%, and the multifactorial nature of the development of marginal ulcers is still not fully understood. Azizullah Beran et al. found that *Helicobacter pylori* was the first factor that led to the development of marginal ulcers and that the mechanism may be the inflammatory response caused by the bacteria leading to gastritis and the eventual development of marginal ulcers (62). Eventually develop into a marginal ulcer. In addition, diabetes can lead to an increased risk of marginal ulcers through mechanisms such as small vessel ischaemia and delayed wound healing (63). However, previous studies have presented some dissenting voices regarding the use of PPIs. Azizullah Beran et al. concluded that the use of PPIs can help to reduce the risk of marginal ulcers, but another study concluded that PPI therapy is not effective in preventing marginal ulcers or their complications (62, 64). Currently, clear criteria for the use of PPIs in marginal ulcers are lacking, and the optimal duration of PPI-based interventions to prevent marginal ulcers is not yet uniform. Future research on marginal ulcers should focus on exploring the pathogenesis and development of medication criteria.

#### 4.2.3 Gut microbiota

The human stomach contains a variety of microorganisms that maintain a delicate balance in the ecological stability of the stomach. However, long-term colonisation by *Helicobacter pylori* triggers an inflammatory response. The altered intragastric environment leads to the disruption of the original balance among microorganisms, thus causing ecological dysregulation of the stomach (65–67). The human gut microbiome is primarily comprised of *Firmicutes*, *Bacteroidetes*, *Actinobacteria*, *Proteobacteria*, and *Verrucomicrobia* (68). Colonisation by *Helicobacter pylori* can cause alterations in the gastric microenvironment and disturbances in the gastric microbiota, but its impact on microbial diversity has not yet been unanimously determined. Some researchers have observed that the microbial diversity in the gastrointestinal tract is lower in patients infected with *Helicobacter pylori* than in the uninfected population (69), whereas others have found that the microbial diversity is higher in patients infected with *Helicobacter pylori* (70), which may be related to the computational techniques and methods used in the statistics (71). The human gastrointestinal tract is characterised by the presence of a large number of commensal microorganisms involved in the immune activities and metabolic homeostasis of the host, and the efficiency of these efforts is closely related to the species and number of commensal microorganisms (72). When the intestinal mucosa recognises a specific microbial receptor, it sends a signal to the immune system. This signal can trigger a protective response in commensal bacteria that manifests as either inflammation or apoptosis. These responses are crucial for the development and regulation of immune homeostasis (68). Disruption of the gastric mucosal barrier is one of the causes of gastric ulcers, and the gastric mucosal layer plays a protective role in the gastric mucosa (73). One study showed that reduction of the mucus layer increases the potential for bacterial translocation and influx of Th17 cells, and that some probiotics can protect the integrity of the gastric mucosal barrier by upregulating prostaglandins and tight junction protein expression, promoting cell proliferation and mucus secretion, and inhibiting apoptosis (74, 75). In addition, colonisation of the gastric mucosa by *Helicobacter pylori* induces a pro-inflammatory response in gastric epithelial cells. Some probiotics can ameliorate this inflammatory response by modulating signalling pathways and cytokines (76, 77). Therefore, appropriate probiotic supplementation can facilitate the treatment of *Helicobacter pylori* infection and provide protection after *Helicobacter pylori* eradication, which has important research implications for the treatment of gastric ulcers.

#### 4.2.4 Antibiotic resistance

As mentioned earlier, *Helicobacter pylori* infection is the main cause of gastric ulcers. Therefore, the use of antibiotics is an important part of the treatment of gastric ulcers. Standard triple therapy to eradicate *Helicobacter pylori* showed good efficacy in the last century (78–80). However, the *Helicobacter pylori* eradication rate has steadily declined in recent years, which is strongly related to the increased resistance of *Helicobacter pylori* to one or more antibiotics (81). Metronidazole and clarithromycin are two antibiotics with key roles in triple therapy, but the emergence of resistance to both antibiotics has been increasing annually worldwide. Therefore, bismuth quadruple therapy and non-bismuth quadruple therapy may be used as alternative therapies for *Helicobacter pylori* eradication in areas with high rates of antibiotic resistance, as well as when the first use of standard triple therapy fails. However, even with multiple *Helicobacter pylori* eradication regimens, increased antibiotic resistance remains a major cause of eradication failure, owing to increased antimicrobial drug resistance and epidemiologic changes (82). Therefore, therapeutic options that avoid the use of antibiotics, such as acupuncture, probiotics, and herbal therapies, are being actively sought for the treatment of gastric ulcers (83). A recent study on acupuncture therapy found that it significantly reduced gastroduodenal injury, decreased the microbial abundance of the thick-walled phylum, and increased the abundance of the anaplasmosis phylum in the stomachs of mice (84). An increase in *Helicobacter pylori*-infected mice with thick-walled bacteria and a decrease in *Mycobacterium avium* in the stomach can cause further damage to the gastric mucosa (85). As an ancient therapeutic tool, herbal therapy has been valued and recognised in the context of the various adverse reactions of conventional medications used in the treatment of gastric ulcers (86). Some studies have suggested that combining herbal and antibiotic therapies may improve *Helicobacter pylori* eradication rates (87). However, owing to the emergence of drug-resistant bacterial strains and the uncertainty of herbal components, herbal therapies should be further researched and screened to ensure the effectiveness and side-effect-free nature of the plants used in the treatment of gastric ulcers or other diseases. In addition, the use of antimicrobial peptides and modulation of oxygen concentration in the gastric environment can eliminate *Helicobacter pylori* without antioxidants (88, 89). These approaches offer valuable research avenues for non-antibiotic treatment of *Helicobacter pylori*.

## 5 Conclusion

This study aimed to provide insights and guidance for advancing gastric ulcer research. Through analysis of two decades’ literature in this field, we examined the progression of gastric ulcer studies and projected research trajectories based on analytical evidence. Keyword clustering identified three research hotspots: ulcer prevention strategies, pathogenesis investigation, and novel therapeutic development. Breaking keywords analysis revealed four emerging focal points: antioxidant applications, marginal ulcer dynamics, gut microbiota interactions, and antibiotic resistance mechanisms. The systematic integration of these emerging focal points with the current research hotspots is expected to drive comprehensive exploration of critical issues while revealing new investigative pathways.

### 5.1 Strengths and limitations

In this study, we utilised CiteSpace software to analyse and visualise gastric ulcer data from 2004 to 2024, summarising key issues and trends in gastric ulcer research. However, the inherent limitations of this study require consideration. First, our analysis was limited to data sourced exclusively from the Web of Science Core Collection (WoSCC), potentially leading to the exclusion of relevant literature from other databases. Second, this study was restricted to English-language sources, which may have resulted in the omission of significant findings and perspectives from non-English-speaking countries. Future studies should aim to address these limitations by incorporating a broader range of databases and including literature in multiple languages.

## Data Availability

The original contributions presented in the study are included in the article/supplementary material, further inquiries can be directed to the corresponding authors.
